# Construction of Individual Morphological Brain Networks with Multiple Morphometric Features

**DOI:** 10.3389/fnana.2017.00034

**Published:** 2017-04-25

**Authors:** Wan Li, Chunlan Yang, Feng Shi, Shuicai Wu, Qun Wang, Yingnan Nie, Xin Zhang

**Affiliations:** ^1^College of Life Science and Bioengineering, Beijing University of TechnologyBeijing, China; ^2^Department of Biomedical Sciences, Cedars-Sinai Medical Center, Biomedical Imaging Research InstituteLos Angeles, CA, USA; ^3^Department of Internal Neurology, Tiantan HospitalBeijing, China

**Keywords:** individual morphological brain network, multiple morphometric features, feature vector, graph theory, reliability

## Abstract

In recent years, researchers have increased attentions to the morphological brain network, which is generally constructed by measuring the mathematical correlation across regions using a certain morphometric feature, such as regional cortical thickness and voxel intensity. However, cerebral structure can be characterized by various factors, such as regional volume, surface area, and curvature. Moreover, most of the morphological brain networks are population-based, which has limitations in the investigations of individual difference and clinical applications. Hence, we have extended previous studies by proposing a novel method for realizing the construction of an individual-based morphological brain network through a combination of multiple morphometric features. In particular, interregional connections are estimated using our newly introduced feature vectors, namely, the Pearson correlation coefficient of the concatenation of seven morphometric features. Experiments were performed on a healthy cohort of 55 subjects (24 males aged from 20 to 29 and 31 females aged from 20 to 28) each scanned twice, and reproducibility was evaluated through test–retest reliability. The robustness of morphometric features was measured firstly to select the more reproducible features to form the connectomes. Then the topological properties were analyzed and compared with previous reports of different modalities. Small-worldness was observed in all the subjects at the range of the entire network sparsity (20–40%), and configurations were comparable with previous findings at the sparsity of 23%. The spatial distributions of the hub were found to be significantly influenced by the individual variances, and the hubs obtained by averaging across subjects and sparsities showed correspondence with previous reports. The intraclass coefficient of graphic properties (clustering coefficient = 0.83, characteristic path length = 0.81, betweenness centrality = 0.78) indicates the robustness of the present method. Results demonstrate that the multiple morphometric features can be applied to form a rational reproducible individual-based morphological brain network.

## Introduction

As a sophisticated but well-organized system, the human brain is capable of processing multiple tasks with high efficiency (Sporns et al., 2004). The interconnected regions of the brain are apt to delineate the operation pattern of integration and segregation, which indicating the ability to rapidly organize specialized information from distributed brain regions functionally, while specialized processing to occur within densely interconnected groups of brain regions (Rubinov and Sporns, 2010; Sporns, 2013). Recently, the brain network has been applied to characterize the effects of age, gender (Damoiseaux et al., 2008; Gong et al., 2009b; Chen et al., 2011; Yan et al., 2011) and psychiatric disorders (Bassett et al., 2008; He et al., 2009; Van Den Heuvel et al., 2010; Alexander et al., 2012; Shi et al., 2013).

Brain networks are generally categorized into three modalities through different neuroimaging and electrophysiological techniques. Functional networks are formed using functional magnetic resonance imaging (fMRI) (Eguiluz et al., 2005; Bassett and Bullmore, 2006), electroencephalogram (EEG) (Micheloyannis et al., 2006; Stam et al., 2007), or magnetoencephalogram (MEG) (Stam, 2004). An anatomical network is built using diffusion tensor imaging (DTI) (Hagmann et al., 2007; Gong et al., 2009a), and a morphological network is constructed using structural MRI (He et al., 2007).

Despite the remarkable progress of studies on functional and anatomical connectomes, the morphological brain network issue was raised relatively late (Evans, 2013). He et al. (2007) used cortical thickness as the regional descriptor to realize the construction of a network by computing the correlations across regions across a large scale; their study is the first to document that the morphological brain network exhibits small-worldness and a scale-free degree distribution. This methodology has also been applied to different descriptors such as regional gray matter volume (Bassett et al., 2008) and surface area (Sanabria-Diaz et al., 2010). Moreover, the morphological brain network can be employed to explore the hierarchical and modular organizations of the cerebral cortex (Bassett et al., 2008; Chen et al., 2011).

Nevertheless, population-based studies on the morphological brain network result in a severe loss of information of inter-individual differences (Kanai and Rees, 2011). Fortunately, several novel approaches have been recently developed to extract structural information directly from T1-weighted MR images and obtain the interregional connectivities for a single subject (Raj et al., 2010; Zhou et al., 2011; Tijms et al., 2012; Kong et al., 2015). For instance, Raj et al. (2010) proposed an individual-based morphological network construction method by using Gibbs probability models. Similarly, in another study, the probability density function of local morphological features was used as the regional descriptor to build the network (Kong et al., 2015). In addition to these mathematical methods, Tijms et al. (2012) employed a cube-based approach. In this approach, after the intensity of voxels is concatenated into a feature vector for each cube, the inter-cube connectivities can be derived individually as the correlation coefficient. Notably, in this method, the size of the node in the brain network has shrunk from the atlas-based region to the voxel-based cube. Furthermore, Batalle et al. (2013) presented a method to normalize the atypical morphological networks, such as the cube partition (Tijms et al., 2012), to standardized brain networks with nodes identified using a parcellation scheme (i.e., AAL brain atlas).

Despite the breakthroughs in research on individual-based morphological brain networks, one particular aspect draws our attention. Previous studies have built their morphological networks by using a single cortical feature, such as cortical thickness or voxel intensity. However, there are multiple morphometric features can be extracted from the brain structural organization. Therefore, we extend previous studies by proposing a novel idea for realizing the formation of individual-based morphological brain network using a combination of multiple morphometric features. In the network construction, the interregional connections were computed as the correlation of feature vectors instead of one feature; each of these vectors comprised nine complementary morphometric features.

To test this idea, we applied it to a sample of 55 healthy participants (24 males) aged 20–29 years old. The network nodes were determined using a brain atlas with multiple anatomical regions of interest. The global and local topological properties of the network were computed in accordance with graph theory. The results of small-world configurations were compared with those of previous studies at a similar sparsity level. Furthermore, the nodal betweenness centrality and the spatial distribution of hubs were thoroughly investigated. Finally, the reproducibility of the method was evaluated.

## Materials and methods

### Participants

Fifty-five right-handed healthy subjects (24 male ages ranging from 20 to 29 with mean = 22.92 and standard deviation = 2.89; 31 female ages ranging from 20 to 28 with mean = 21.71 and standard deviation = 2.19) were selected from the Open Access Series of Imaging Studies Database (http://www.oasis-brains.org/). All the subjects were questioned about their medical histories and use of psychoactive drugs before a trained physician began image acquisition. For details on the clinical and demographic information of the subjects, please refer to Marcus et al. (2007).

### Image acquisition

For each subject, three or four individual T1-weighted magnetization-prepared rapid gradient-echo images were acquired using a single Siemens 1.5 T Vision scanner with the following parameters: repetition time = 9.7 ms, echo time = 4.0 ms, inversion time = 20 ms, flip angle = 10°, sagittal orientation with 128 slices, and resolution = 1 × 1 × 1.25 mm. Multiple T1 images obtained for each subject were motion-corrected and then averaged to achieve an improved signal-to-noise ratio. For additional details on the post-processing information regarding the raw images, please refer to Marcus et al. (2007).

### Measurements of multiple morphometric features

**Figure 1:**
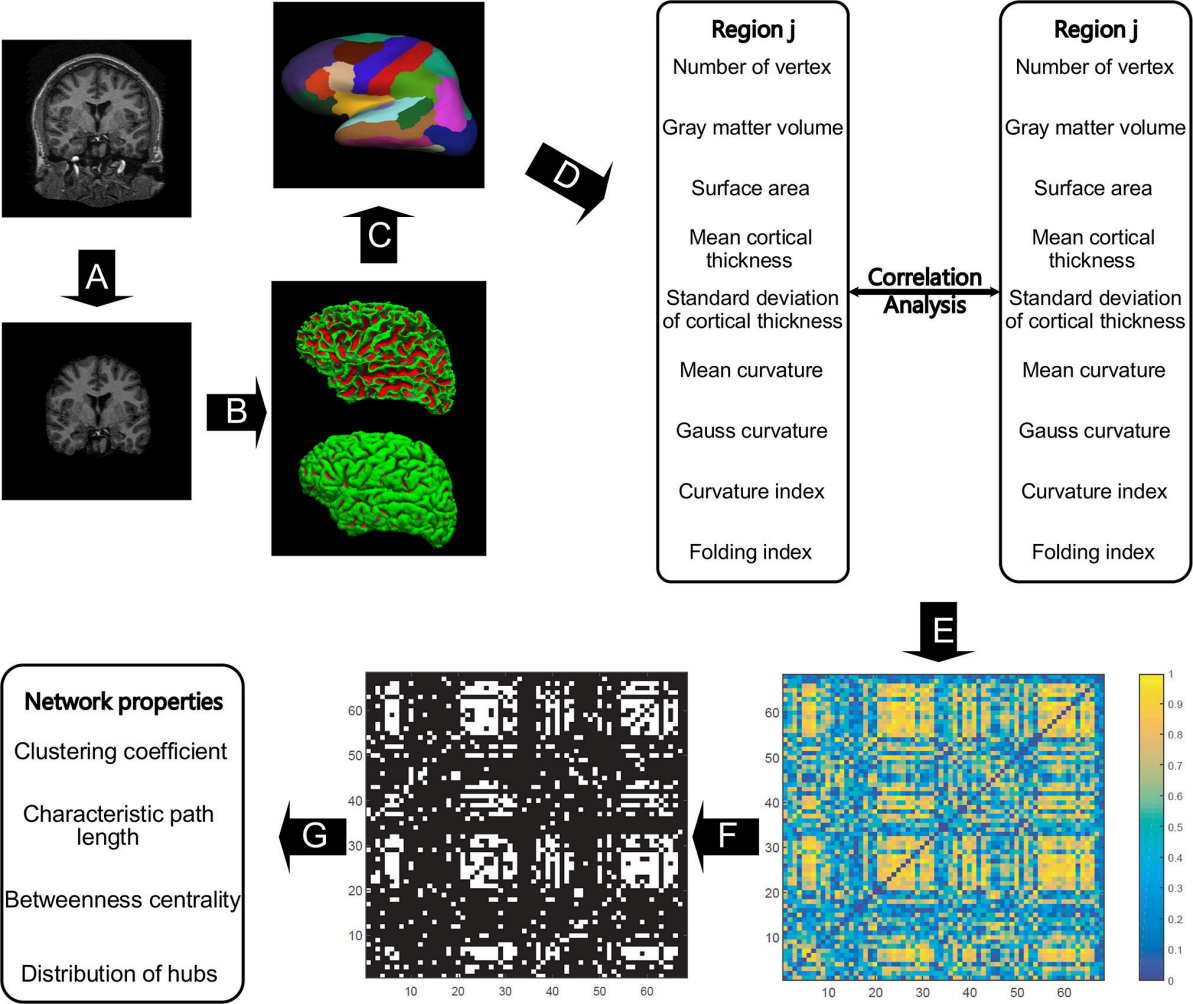
**The general flowchart of the individual-based morphological brain network construction and analyses**. The steps from **(A–D)** were accomplished using FreeSurfer. Intensity bias was corrected, and the skull was stripped **(A)** before the inner and outer surfaces of gray matter were extracted **(B)**. All the involved morphometric features were first measured between inner and outer surfaces **(C)**, and then the feature results were mapped to the Desikan–Killiany cortical atlas to obtain the regional feature measurements **(D)**. For each region, nine morphometric features were concatenated as a feature vector to obtain the correlation coefficient between any two regions to frame the individual-based morphological connectivity **(E)**. The connection matrix was then repeatedly binarized based on the sparsities (from 20 to 40% with a step size of 1%) to generate the network graphs **(F)**, and then the network topological properties were analyzed in accordance with graph theory **(G)**.

All the images were preprocessed through FreeSurfer (version 5.3.0), which can be freely downloaded online (http://surfer.nmr.mgh.harvard.edu/). Studies have assessed FreeSurfer's performance with images acquired from various MRI scanners or sequences and received reliable results for cortical and subcortical measurements (Khan et al., 2008; Shi et al., 2013; Mulder et al., 2014). Details on FreeSurfer's processing pipeline can be found in Dale et al. (1999) and Fischl et al. (1999). In short, the raw images were resampled into 256 × 256 × 256 isotropic resolutions with a voxel size of 1 × 1 × 1 mm. The intensity bias was subsequently corrected in a volume-based stream, and the skull was stripped, followed by volumetric labeling and white matter segmentation (Figure 1A). In the surface-based stream, the surface of white matter was extracted as the inner surface, and then nudged to the gray–pial interface to generate the outer surface (Figure 1B). The FreeSurfer surface was created as a mesh with a non-uniform grid, also known as a vertex. Hence, the cortical morphometric features can be measured using every vertex between the inner and outer surfaces (Figure 1C). Finally, the built-in Desikan-Killiany cortical atlas (Desikan et al., 2006) was applied to obtain the regional measurements (Figure 1D). This atlas presents the parcellation of 34 regions for each hemisphere on the basis of the structural patterns of the gyrus and sulcus (Supplementary Table 1).

Nine morphometric features were initially employed in this study (Figure 1E): (1) the number of vertices in each region, (2) regional gray matter volume, (3) surface area, (4) mean cortical thickness, (5) standard deviation of cortical thickness, (6) mean curvature, (7) Gauss curvature, (8) curvature index, and (9) fold index. In particular, the first two features are tallied as the sum of the vertices and voxels in a region, respectively. Surface area was computed as the total of the areas of all vertices falling within a region (Panizzon et al., 2009). The cortical thickness is measured as the distance between the inner and outer cortical surfaces at each vertex (Fischl et al., 1999). The curvature is calculated as the reciprocal of the radius of the inscribed circle for each vertex, and the signs of the gyrus and sulcus are opposite (Li et al., 2014). The Gaussian curvature of a surface at a given point is the product of the principal curvatures, which are the eigenvalues of the shape operator at the point (Pienaar et al., 2008). They measure how the surface bends by different amounts in different directions at that point. The folding index is calculated as the number of cortices buried within the sulcal folds, as compared with the number of cortices on the visible outer cortex (Schaer et al., 2008). Notably, all the surface computations were performed in the native space, enabling the above-mentioned features to be measured without deformation.

### Individual-based morphological network construction

To form the network, the regional descriptor was defined as a feature vector, which is the concatenation of the nine morphometric features in each region, as shown in Figure 1. Therefore, the interconnected matrix is generated by computing the Pearson correlation coefficient for each pair of feature vectors (Figure 1E). Significant differences exist between the orders of magnitude of each morphometric feature (10^−2^ to 10^3^). Thus, the z-score values were computed for each feature as the standardized values before the correlation calculation. The z-score values were calculated from each morphometric feature by first subtracting the mean of all values from each individual value and then dividing each remainder by the standard deviation of all the values, as realized by the normalization function in the statistical analysis software SPSS v22.0 (SPSS Inc., Chicago IL, USA). The correlation matrix was then binarized to generate the unweighted and undirected networks (Figure 1F), because this matrix captures the underlying anatomical connection patterns, while providing a simple method for investigating the network (He et al., 2007). To maintain both positive and negative high correlations, the absolute values of matrixes were applied for binarization. Lastly, such network properties as small-world configurations and definition of hubs (Figure 1G) were analyzed in accordance with graph theory.

For binarization, sparsity was used to express the extent of thresholding, which is the ratio of existing connections to the total possible ones. However, the appropriate sparsity remains an open question (Zhu et al., 2012). Therefore, the network properties were evaluated as a function of sparsity ranging from 20 to 40% with a step size of 1%. The range was determined using the requirements stating that the minimum sparsity guarantees no isolated node in the network, and the maximum one ensures small-worldness. The other advantage of using a certain range of sparsity is that it allows us to investigate the network properties at the same level for all the subjects, as the same number of existing connections are found in each case. Additionally, the sparsity of 23% was highlighted to enable direct comparison with previous studies.

### Analyses of network properties

The network properties were computed using the Brain Connectivity Toolbox (BCT) (Rubinov and Sporns, 2010) and Graph-theoretical Network Analysis (GRETNA) toolkit (Wang et al., 2015). SPSS was used for the single-feature normalization (see individual-based morphological network construction) and all the statistical analyses, while the spatial distribution of hubs was visualized using the BrainNet Viewer toolkit (Xia et al., 2013). The multiple comparison was corrected using the false discovery rate (FDR) proposed by Genovese et al. (2002).

#### Small-world configurations

In 1998, Watts and Strogatz first proposed the notion of a small-world network, which exhibits a similar characteristic path length (*L*_*p*_) but a higher clustering coefficient (*C*_*p*_) than a random network (Watts and Strogatz, 1998). In particular, *C*_*p*_ represents the average of the clustering coefficients of all the nodes (*C*_*i*_) in a network, where *C*_*i*_ expresses the likelihood that the neighborhoods of the nodes are connected. Specifically, *C*_*i*_ of a given node is computed as the proportion of connections among its neighbors which are actually realized compared with the number of all possible connections. Thus, *C*_*p*_ quantifies the extent of local cliquishness or the efficiency of a particular network's information transfer (Latora and Marchiori, 2001). In addition, *L*_*p*_ represents the characteristic path length of the graph, which is the average shortest path length among all pairs of nodes in the network. However, correct calculation of *L*_*p*_ based on the definition is unachievable because not every pair of nodes in a binary brain network is connected. To avoid this situation, *L*_*p*_ is measured as the “harmonic mean” distance between pairs of nodes proposed by Newman (2004). *L*_*p*_ quantifies the parallel information propagation ability, or the global efficiency of a network (Latora and Marchiori, 2001). Therefore, small-worldness can be demonstrated mathematically as

$$\gamma =\frac{C_{p}^{observed}}{C_{p}^{random}}\;>1\text{ and }\lambda =\frac{L_{p}^{brain}}{L_{p}^{random}}\;\approx \;1$$

or merged into one formula

$$\sigma =\frac{\gamma }{\lambda }>1,$$

where the corresponding random networks consist of the same number of nodes and edges (Achard et al., 2006).

#### Spatial distribution of hubs

The betweenness centrality (BC) is defined as the number of shortest paths between any two nodes running through the given node, and measures the nodal ability of information flow throughout the network (Freeman, 1977). In particular, two considerations are adopted to determine the hubs. The first specifies the hubs for each subject; thus, the average of BC for the entire sparsity range (mspBC) is used to represent nodal BC for each subject. The next consideration involves realizing the identification of hubs at each sparsity; hence, the mean BC of all the subjects (msjBC) was employed to represent nodal BC at each sparsity. Overall, the hubs were selected with the same criterion, indicating that the nodes should achieve a higher BC (mspBC or msjBC) than the sum of the mean and standard deviation for the entire network, as demonstrated below.

$$BC\;>\;mean_{BC}\;+\;standard\;deviation_{BC}$$

The nodal degree was not used to determine the hubs because in contrast with BC, this degree only measures the connections linked with the node, rather than the shortest path.

### Reproducibility

To evaluate the reproducibility of morphometric features and the present method, the intraclass correlation coefficient (ICC) was applied. ICC was first defined by Mcgraw and Wong (1996) as the fraction of the variance of chosen graphic property between subjects to the total variance, which is the summation variance of between and within subjects of that property.

$$ICC=\frac{\sigma_{between}^{2}}{\sigma_{between}^{2}+\sigma_{within}^{2}}$$

If the measurements of repeated scans are consistent for each subject, the ICC would be close to one. An ICC value above 0.75 is considered excellent, and one ranging from 0.6 to 0.75 is considered good (Bennett and Miller, 2010). ICC was computed using the “Reliability Analysis” function of SPSS.

## Results

Before the formation of networks, nine of the morphometric features were firstly measured of its robustness by SPSS. The mean value of the entire brain was used to realize the ICC measurement. The number of vertices (ICC = 0.95 with *p* = 3.1 × 10^−4^), mean curvature (ICC = 0.80 with *p* = 2.4 × 10^−2^), surface area (ICC = 0.99 with *p* = 7.3 × 10^−6^), volume (ICC = 0.96 with *p* = 1.6 × 10^−4^) and standard deviation of thickness (ICC = 0.94 with *p* = 6.3 × 10^−4^) are significantly reliable. Robustness was also found in Gauss curvature (ICC = 0.68) and mean thickness (ICC = 0.73), but the results are less significant with *p*-value of 7.6 × 10^−2^ and 5.3 × 10^−2^, separately. However, the index of fold and curvature are failed to present acceptable reproducibility (ICC = 0.73 with *p* = 0.76 and ICC = 0.36 with *p* = 0.29, respectively). Therefore, only seven of morphometric features were employed in the network construction (Figure 1E, number of vertices, mean and Gauss curvature, surface area, volume, mean and standard deviation of thickness). The subsequent network analyses included the following: (1) assessment of small-world configurations (*C*_*p*_, *L*_*p*_, γ, λ, and σ) of the networks, (2) investigation of BC and spatial distribution of the networks' hubs, and (3) estimation of the method's reproducibility. For a rational demonstration, the sparsity applied ranged from 20 to 40% with a step size of 1% to extract all of the above-mentioned measurements for each individual-based brain network.

### Small-world configurations

**Figure 2:**
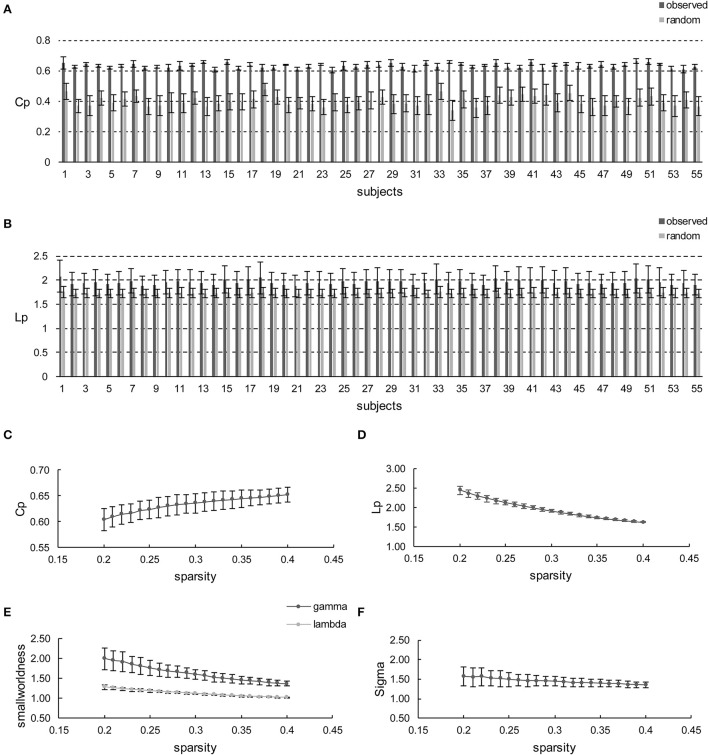
**Small-world configurations of the individual-based morphological networks. (A,B)** Represent the average *C*_*p*_ and *L*_*p*_ of sparsities for each subject. The error bar indicates the deviation caused by different sparsities. **(C–F)** Show the average *C*_*p*_, *L*_*p*_, γ, λ, and σ of the subjects for each sparsity (from 20 to 40% with a step size of 1%). The error bar indicates the deviation caused by different subjects.

Initially, apart from the subjects' respective brain networks, *C*_*p*_ and *L*_*p*_ were also derived from the corresponding random networks at each sparsity for each subject. Hence, the comparisons were conducted between the different subjects' brain networks and random networks (Figures 2A,B) or sparsities (Figures 2C–E). The comparison shows that *C*_*p*_ and *L*_*p*_ are profoundly higher than the random ones all over the sparsity range with the maximum *p* = 1.14 × 10^−11^ (*t*-values ranged from 11.75 to 27.77 with FDR correction) and *p* = 6.15 × 10^−3^ (*t*-values ranged from 2.98 to 5.00 with FDR correction), as revealed using the independent two-sample *t*-test (Figures 2A,B, respectively). Likewise, γ was larger than one (max = 1.99, min = 1.37) throughout the whole sparsity range, while λ was close to one (max = 1.28, min = 1.02). Hence, as expected σ are found larger than one throughout the entire sparsity range (max = 1.56, min = 1.35) which confirms the existence of small-worldness. Overall, the individual networks exhibit significantly higher *C*_*p*_ than the random network, while remaining similar *L*_*p*_. Thus, small-worldness is confirmed for each subject at every sparsity. Moreover, the increase and decrease of small-world configurations, as sparsity increased, is illustrated in Figure 2C. Such small-world configuration fluctuations are in accordance with the variation tendency documented in previous reports (He et al., 2007; Kong et al., 2015).

Furthermore, the sparsity of 23% is highlighted for convenient comparison with previous studies, including those based on individual-based morphological brain networks (Tijms et al., 2012; Kong et al., 2015), those involving population-based morphological brain networks with various regional descriptors (He et al., 2007; Sanabria-Diaz et al., 2010; Zhu et al., 2012), and functional brain network studies (Zhang et al., 2011). As listed in Table 1, the population-based morphological networks and functional network exhibit smaller results than the current findings, whereas the individual-based morphological network studies present relatively similar results, particularly that of Kong et al. (2015). This finding suggests that the individual morphological brain networks may demonstrate a stronger integration and segregation because the inter-individual variability is highly reserved (Kanai and Rees, 2011).

**Table 1:** **Comparison of small world configurations between the present study and previous studies**.

**Study**	**Descriptor**	***N***	***C_*p*_***	***L_*p*_***	**γ**	**λ**	**σ**	***s* (%)**
**INDIVIDUAL-BASED MORPHOLOGICAL BRAIN NETWORK**								
Present study	Multiple morphometric features	68	0.62	2.23	1.81	1.22	1.52	23
Kong et al., 2015	Probability density functions	90	0.66	1.92	1.74	1.15	1.50	23
Tijms et al., 2012	Voxel intensity	6,982	0.53	1.86	1.35	1.05	1.28	23
**POPULATION-BASED MORPHOLOGICAL BRAIN NETWORK**								
He et al., 2007	Cortical thickness	54	NR	NR	≈1.5	≈1.15	≈1.3	23
Zhu et al., 2012	Gray matter volume	90	≈0.26	NR	≈1.20	≈1.03	≈1.17	23
Sanabria-Diaz et al., 2010	Surface area	82	≈0.3	≈1.81	NR	NR	≈1.28	22
	Cortical thickness	82	≈0.29	≈1.81	NR	NR	≈1.23	22
**FUNCTIONAL BRAIN NETWORK**								
Zhang et al., 2011	Resting state (0.01 ~ 0.1Hz)	90	≈0.33	≈1.65	≈1.3	≈1	≈1.4	23

*N, C_p_, and L_p_ denote the number of nodes in the networks, the average clustering coefficient and the mean of characteristic path length, respectively. γ represents the ratio of the networks' clustering coefficient and the clustering coefficient of the random network. λ stands for the ratio of the average characteristic path length of the network and the average characteristic path length of the random network. σ shows the small-worldness. NR is “not reported.” The small world attributes of previous studies are referred (without ≈) or inferred (with ≈) at the sparsity of 23%. There are also relevant references, but these are unlisted because the values of these properties are unknowable at the sparsity of 23%. Such references include the studies of Lo et al. (2010) and Gong et al. (2009a) for the anatomical brain network, Liu et al. (2008) for the functional brain network, and **?** for the morphological one*.

### Spatial distribution of hubs

**Figure 3:**
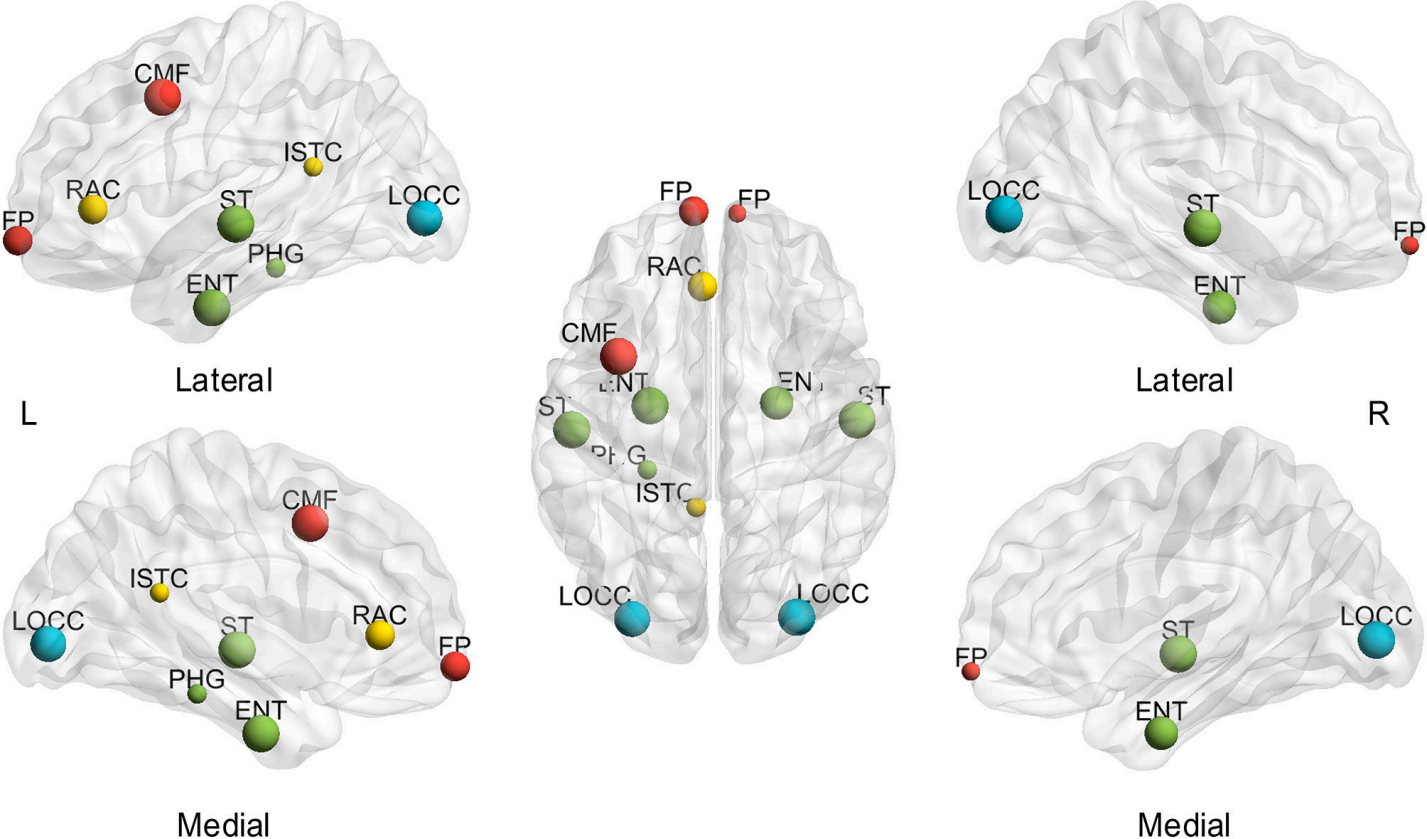
**Hubs with a sparsity range exceeding 20%, based on the average of subjects**. L, left; R, right; A, anterior; P, posterior; CMF, caudal middle frontal gyrus; ENT, entorhinal cortex; FP, frontal pole; ISTC, isthmus cingulate; LOCC, lateral occipital gyrus; RAC, rostral anterior cingulate; PHG, parahippocampal; ST, superior temporal gyrus. The size of the node represents the proportion of sparsity; the largest node denotes 100% of the proportion. The different color of nodes denotes the different lobes: red-frontal lobe; green-temporal lobe; blue-occipital lobe; yellow-cingulate.

In addition to the assessment of small-world configurations, the spatial distribution of hubs was investigated for different subjects and sparsities. First, the effect of sparsities on hub identification was computed. The BC was averaged through all the subjects at each sparsity to investigate the extent of the sparsity range for each hub region (msjBC). A total of ten regions identified as hubs achieved a sparsity range of over 20% (Figure 3), including the bilateral entorhinal cortex (left 100.00%, right 85.71%), superior temporal gyrus (left 100.00%, right 100.00%), lateral occipital gyrus (left 95.24%, right 100.00%), frontal pole (left 71.43%, right 28.57%) and left caudal middle frontal gyrus (100.00%), left rostral anterior cingulate cortex (71.43%), left isthmus cingulate cortex (33.33%), left parahippocampal gyrus (33.33%). In addition, a total of 4 regions were regarded as hubs in a sparsity range of 4.76 to 14.29%, and the rest of the 52 regions were never observed presenting a hub at any sparsity. The specific extent of sparsity range for each node identified as a hub may be found in Supplementary Table 2.

**Figure 4:**
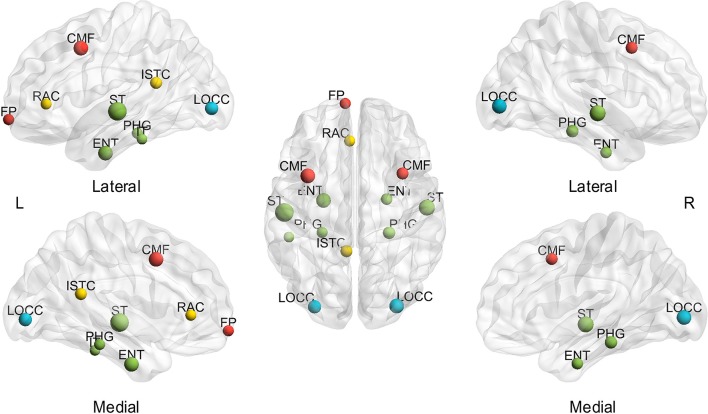
**Hubs in over 20% of subjects based on the average of sparsities**. L, left; R, right; A, anterior; P, posterior; CMF, caudal middle frontal gyrus; ENT, entorhinal cortex; FP, frontal pole; ISTC, isthmus cingulate; IT, inferior temporal gyrus; LOCC, lateral occipital gyrus; RAC, rostral anterior cingulate; PHG, parahippocampal; ST, superior temporal gyrus. The size of the node represents the proportion of subjects; the largest node denotes 54.55% of the proportion. The different color of nodes denotes the different lobes: red-frontal lobe; green-temporal lobe; blue-occipital lobe; yellow-cingulate.

Thereafter, the influence of individual differences on hub determination was investigated. Likewise, the BC was averaged throughout the sparsity range for each subject to determine the number of subjects presenting each region as a hub (mspBC). A total of 14 nodes are hubs for over 20% of the subjects (Figure 4), including the superior temporal gyrus (left 54.55%, right 43.64%), caudal middle frontal (left 38.18%, right 27.27%), entorhinal cortex (left 38.18%, right 25.45%), lateral occipital gyrus (left 32.73%, right 38.18%), parahippocampal gyrus (left 23.64%, right 29.09%), and left isthmus cingulate cortex (27.27%), left rostral anterior cingulate cortex (23.64%), left frontal pole (23.64%), left inferior temporal gyrus (21.82%). A total of 53 regions are considered hubs in 1.82 to 18.18% of the subjects, and just one region comprised no subjects. The specific number of subjects presenting each node as a hub is found in Supplementary Table 3.

**Figure 5:**
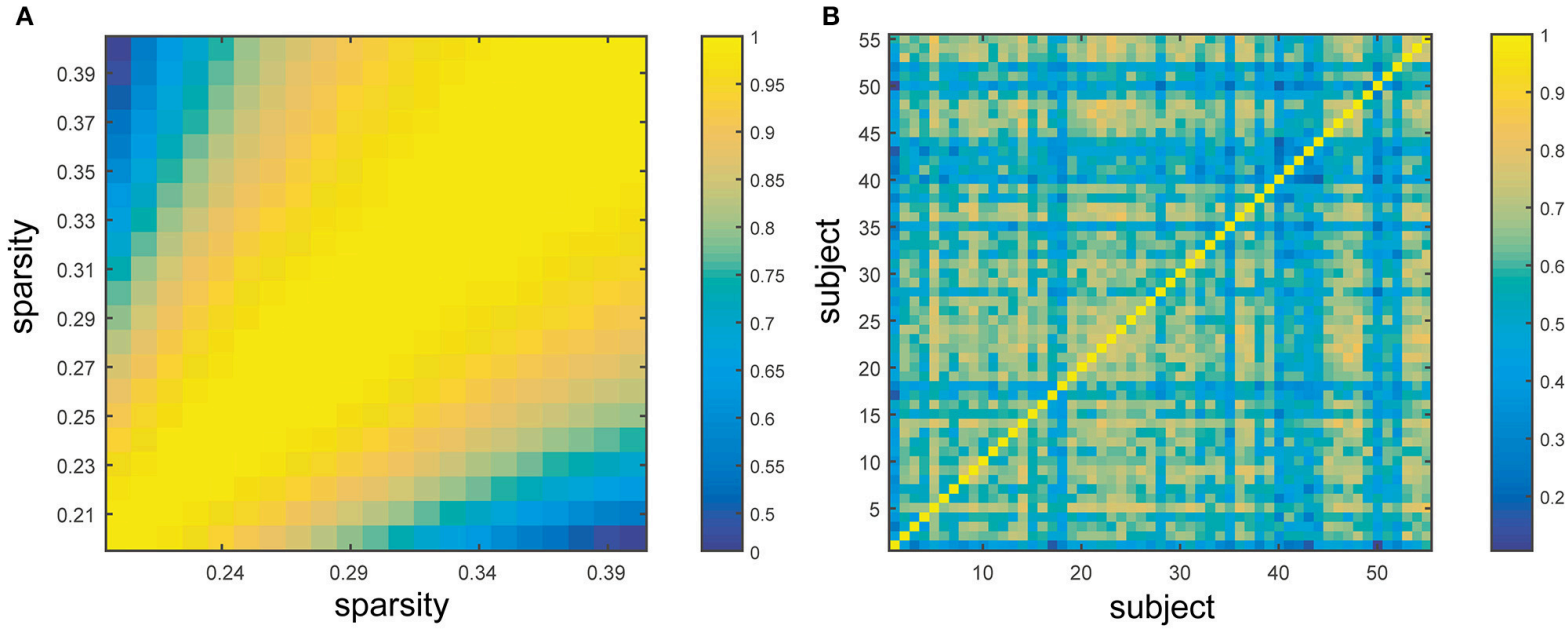
**Similarity across sparsities and subjects. (A)** Represents the similarity between different sparsities from 20 to 40% with the step size of 1%. **(B)** Exhibits the similarity between different subjects. The color bar shows the extent of similarity with blue as dissimilar and yellow as similar.

The similarity of BC across subjects and sparsities was evaluated through the proximity matrix generated by SPSS (Figure 5). As illustrated in the figure (yellow: similar, blue: dissimilar), the inter-subject and inter-sparsity similarities of BC were observed, and the highly comparable BC was found in adjacent sparsities (close to diagonal) (Figure 5A). The individual differences in subjects significantly influence the BC value (Figure 5B). Clearly, the similarities were significantly high across sparsities. These findings are also in agreement with the above-mentioned results, which state that the hubs can be mostly retained for different sparsities, while hub identification varied profoundly for different subjects.

**Figure 6:**
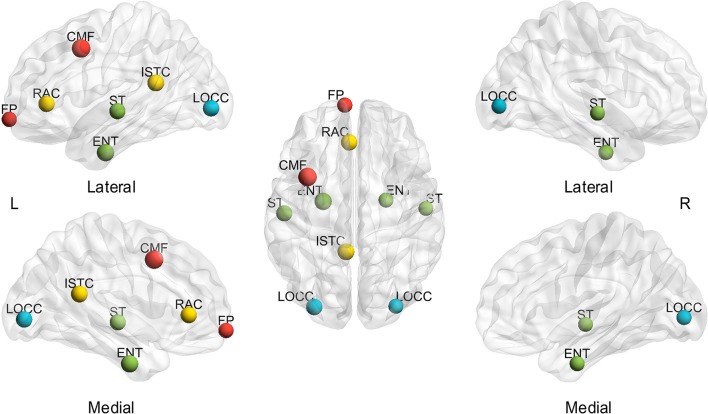
**Spatial distribution of hubs identified based on the average of sparsities and subjects**. L, left; R, right; A, anterior; P, posterior; CMF, caudal middle frontal gyrus; ENT, entorhinal cortex; FP, frontal pole; ISTC, isthmus cingulate; LOCC, lateral occipital gyrus; RAC, rostral anterior cingulate; ST, superior temporal gyrus. The size of the node represents the value of BC; the largest node denotes 107.22 of BC. The different color of nodes denotes the different lobes: red-frontal lobe; green-temporal lobe; blue-occipital lobe; yellow-cingulate.

Furthermore, Figure 6 shows the visualization of hub distribution based on the average BC of all the subjects and the entire sparsity range. The results indicate that ten hubs were identified, comprising six heteromodal or unimodal associative regions and four paralimbic regions (Table 2). All these hub regions have been reported in at least one previous study.

**Table 2:** **The hubs distribution based on the average BC of all the subjects and the entire sparsity range**.

**Regions**	**BC**	**Class**	**References**
Left superior temporal gyrus	107.22	Association	[f1] [f2] [m3]
Right superior temporal gyrus	97.84	Association	[f1] [f2] [m3]
Right lateral occipital gyrus	93.92	Association	[f1] [f3] [m4] [a1]
Left entorhinal cortex	90.48	Paralimbic	[f1] [m1] [a2]
Left caudal middle frontal gyrus	89.61	Association	[f1] [f2] [m3] [m4] [m5]
Left lateral occipital gyrus	88.73	Association	[f1] [f3] [m4] [a1]
Left rostral anterior cingulate	81.03	Paralimbic	[m2]
Right entorhinal cortex	79.00	Paralimbic	[f1] [m1] [a2]
Left frontal pole	79.00	Association	[a3] [f4]
Left isthmus cingulate	77.34	Paralimbic	[f2] [m4]

*All the regions were listed in descending order according to their BC, which is averaged across subjects and sparsities. The classes were defined by Mesulam (1998). The abovementioned references include morphological brain network studies: [m1] Kong et al. (2015), [m2] Bassett et al. (2008), [m3] Wu et al. (2012), [m4] He et al. (2007), [m5] Tijms et al. (2012); anatomical brain network studies: [a1] Gong et al. (2009a), [a2] Harriger et al. (2012), [a3] Hagmann et al. (2008); functional brain network studies: [f1] Achard et al. (2006), [f2] Buckner et al. (2009), [f3] Wang et al. (2010), [f4] Honey et al. (2007)*.

### Reproducibility

The method's reproducibility was evaluated by measuring the ICC index of network properties for scans with acquisitions of two different time points in the same subjects. The ICC was investigated throughout the entire sparsity range. Only *C*_*p*_, *L*_*p*_, and BC were examined in this study because of the instability of random network generation processing.

**Figure 7:**
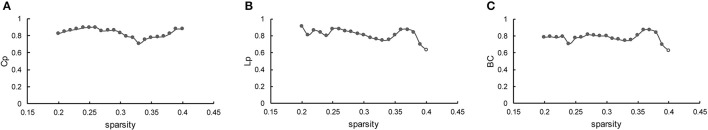
**Reproducibility of the method. (A–C)** Represents the ICC of *C*_*p*_, *L*_*p*_, and BC as a function of sparsity, respectively. The hollow dot indicates the insignificant result.

The results indicated that *C*_*p*_ was highly reproducible (minimum ICC = 0.71, average ICC = 0.83), as plotted in Figure 7A. Moreover, the robustness of *L*_*p*_ (minimum ICC = 0.63, average ICC = 0.81) was fairly stable (Figure 7B), as the similarities to the BC (minimum ICC = 0.629, average ICC = 0.78) are shown in Figure 7C. Most of the results are significant, except for *L*_*p*_ and BC at sparsity of 40% (*p* = 5.6 × 10^−2^ and *p* = 5.7 × 10^−2^, separately). Hence, the reliability of the present method is well-documented in accordance with the standard proposed by Bennett and Miller (2010). The specific value of ICC for the three measurements across the entire sparsity range is shown in Supplementary Table 4.

## Discussions

To our knowledge, this study is the first to collectively employ multiple morphometric features to evaluate the individual morphological brain network. First, small-worldness is confirmed at each sparsity (20%–40% with a step size of 1%) for all the subjects by investigating *C*_*p*_, *L*_*p*_, γ, λ, and σ (Figure 2). In addition, the small-world configurations are found to be compatible with previous reports at the sparsity of 23% (Table 1). Thereafter, subject diversity was assessed and was found to significantly influence hub identification, whereas the influence of variety between sparsities was shown to be minimal (Figures 3–5). In addition, the hubs determined by the average of the entire sparsity range and population were found to be highly consistent with previous findings (Figure 6 and Table 2). Finally, the method's reproducibility was ascertained by verifying the ICC of *C*_*p*_, *L*_*p*_, and BC throughout the sparsity range (Figure 7). Overall, the results suggest that the proposed method may offer a new way to build the morphological brain network individually with multiple morphometric features.

### Interregional morphological similarity

The interregional morphological similarity has been observed and verified repeatedly in recent studies based on such morphometric features as cortical thickness and regional volume (Lerch et al., 2006; He et al., 2007; Bassett et al., 2008; Sanabria-Diaz et al., 2010). The interpretation of this observation implies that the brain regions exhibit covaring morphometric features that can form the connected structure, which may capture long-term neurobiological effects (Mechelli et al., 2005). However, the underlying mechanism of this covariance pattern remains elusive. Some conjectures have been debated in the scientific literature, including mutually trophic effects (Ferrer et al., 1995; Aid et al., 2007), environment-related plasticity (Maguire et al., 2000; Draganski et al., 2004; Mechelli et al., 2004), genetic influence (Schmitt et al., 2008), and normal development (Raz et al., 2005; Chen et al., 2011). The axonal tension theory has also been raised recently (Tijms et al., 2012; Kong et al., 2015), stating that the interconnected areas are becoming either thicker or thinner as a result of being pulled by a mechanical force (Van Essen, 1997).

Moreover, the relationships among morphological (M), functional (F), and anatomical (A) connectivities were investigated in previous studies. For instance, Reid et al. (2017) have demonstrated that the extent of accordance between F-M modalities varied remarkably across seed regions. Wang et al. (2015) have illustrated a tight coupling of F-M modalities in connectivity strength and network topologic organizations (i.e., modules, rich club, and motifs), further indicating that the neuropsychiatric disorders may considerably break this coupling. In addition, Gong et al. (2012) have found an A-M convergence in 35–40% of morphological connectivities, with the majority of the convergences observed in the positive morphological connectivities.

Further research in neurobiology would significantly assist the construction and exploration of morphological brain networks. In addition, the data on different modalities were typically used or analyzed separately because they were supposed to capture the distinct underlying phenomena. However, the correspondences in topological structures have been recently observed across modalities, suggesting that the combination of multimodal research will become increasingly important in neuroscience.

### Spatial distribution of hubs

The spatial distribution of hubs (nodes with higher BC of average subjects and the entire sparsity range) in the present study was found to be strikingly similar to the results of functional studies. The consistent regions are the bilateral superior temporal gyrus, entorhinal cortex, and lateral occipital gyrus, and left isthmus cingulate, frontal pole (Table 2). Such accordance is quite thought-provoking, although the different criteria were employed to identify the hubs (BC, degree, and *L*_*p*_). Hence, the findings may suggest that the individual-based morphological brain networks may exhibit an improved consistency with functional studies.

In addition, the hub spatial distribution of different subjects (averaged by sparsities) shows striking diversity in the present study (Supplementary Table 3). As illustrated in Figures 4, 5 (right), inter-individual variability is implied as a major influence on hub determination. Indeed, a number of studies have demonstrated unmistakable discrepancies in brain structures across individuals (Kanai and Rees, 2011). For example, genetic differences can lead to brain morphological changes (Thompson et al., 2001; Pol et al., 2006), as well as the variance in occupations (Maguire et al., 2000; Gaser and Schlaug, 2003) and skills (Mechelli et al., 2004). Moreover, cognition is found to be correlated with cerebral structures. For instance, individual differences in degrees of empathy were observed to be associated with gray matter density (Eres et al., 2015) and volume (Banissy et al., 2012). Psychiatric diseases such as epilepsy also involve unpredictable foci locations and numbers (Engel, 2006). Therefore, individual-based investigations are indispensable in clinical and scientific research.

Hence, investigation of the organization of morphological brain networks, along with functional and anatomical network organization on a case-by-case basis is both intriguing and indispensable. The individual-based accordance across modalities may also be an indicator in future cognitive and psychiatric research.

### Methodology issues and future research

Several methodological issues are found in the present study, which should be addressed and solved in future research.

First, the interregional connections were measured as the Pearson correlation coefficient in the present study, as in many earlier studies (He et al., 2007; Chen et al., 2011; Shi et al., 2013). However, this measurement shows that the observed connections between any two regions are actually the summation of the direct and indirect correlations. A widely used approach for eliminating the influence of other regions is to alternatively use partial correlation (Bassett et al., 2008). The number of variables should be less than a number of samples of each variable in partial correlation computation. However, in the present study, a total of 68 regions (i.e., variables) with nine morphometric features (i.e., samples) are used, which goes against the regular partial correlation. Consequently, in future research, the partial correlation computation must be adjusted to increase the accuracy of results addressing interregional connections.

Second, the networks were binarized with a sparsity range from 20 to 40% for topological analyses, as in previous studies (He et al., 2007; Kong et al., 2015). This binarization leads to the exclusion of 60–80% of connection information simply because such connections were deemed “not important” based on the correlation algorithms. The underlying mechanism of morphological connections remains unclear (see interregional morphological similarity). Therefore, whether or not the “not important” connections are really useless has not been confirmed (Barrat et al., 2004). As such, further exploration of brain networks as the full-connected weighted graph is essential to obtaining additional insights into the human brain.

Third, the nine morphometric features applied in the present study were far from painstakingly selected. However, the anatomical information including cortical thickness, gray matter volume, surface area, and curvature is varied (He et al., 2007; Sanabria-Diaz et al., 2010). In addition, redundant information may have existed in the three curvature-related measurements (mean curvature, Gauss curvature, and curvature index). Therefore, an optimal selection of morphometric features will be further explored.

Fourth, the present study only investigated the individual-based morphological connectivities, whereas the functional and anatomical connections were found to demonstrate a mutual relationship with the morphological connections in the present and previous studies (Sui et al., 2012; Mueller et al., 2013). Hence, in future research, the multimodal images should be employed in individual-based brain network analyses.

## Conclusion

In this study, a new idea is proposed to construct an individual-based morphological brain network. To our knowledge, this study is the first to collectively use multiple morphometric features to form interregional connections. The results of network topological analyses have demonstrated this method's feasibility, and the verification of reproducibility has supported its excellent robustness. Our findings on hubs' spatial distribution have provided profound indications of individual differences that cannot and should not be overlooked. Moreover, the hubs obtained by averaging across subjects and sparsities have been shown to be largely compatible with an individual-based functional study, leading us to investigate whether individual-based multimodal brain networks share further similarities. The proposed method may offer a novel approach in investigating the cerebral organization individually. The interrelationship between modalities, combined with functional connectivities, is worthy of further exploration.

## Ethics statement

This study was carried out with the data of human subjects from the Open Access Series of Imaging Studies database, which is made available by the Washington University Alzheimer's Disease Research Center, Dr. Randy Buckner at the Howard Hughes Medical Institute (HHMI) at Harvard University, the Neuroinformatics Research Group (NRG) at Washington University School of Medicine, and the Biomedical Informatics Research Network (BIRN).

## Author contributions

The design of the work was proposed by WL and CY. The data processing was completed by WL, YN, and XZ. Drafting of the work was completed by WL and CY. Revision of the draft was conducted under the tutelage of FS. SW and QW provided the equipment and medical suggestions for the work. All the authors offered final approval for the version to be published. All the authors agreed to be accountable for all aspects of the work in ensuring that questions related to the accuracy or integrity of any part of the work are appropriately investigated and resolved.

## Funding

This research was partly supported by Beijing Nova Program (xx2016120), National Natural Science Foundation of China (81101107, 31640035 and 71661167001), Natural Science Foundation of Beijing Municipality (4162008), and Beijing Municipal Education Commission (PXM2017_014204_500012).

### Conflict of interest statement

The authors declare that the research was conducted in the absence of any commercial or financial relationships that could be construed as a potential conflict of interest.
