# Supplementary material for: Construction of Individual Morphological Brain Networks with Multiple Morphometric Features
Source: Front Neuroanat. 2017 Apr 25;11:34. doi: 10.3389/fnana.2017.00034 (PMC5403938; doi:10.3389/fnana.2017.00034)
Supplement: Supplementary file 2 [file Table2.DOCX]

Table 2. The extent of sparsity range for each node as a hub. The bold denotes the node identified as a hub for over 20% of sparsity range.

| **Regions** | **Extent of sparsity range** | **Regions** | **Extent of sparsity range** |
| --- | --- | --- | --- |
| BSTS_L | 0.00% | BSTS_R | 0.00% |
| CAC_L | 0.00% | CAC_R | 0.00% |
| **CMF_L** | **100.00%** | CMF_R | 9.52% |
| CUN_L | 0.00% | CUN_R | 0.00% |
| **ENT_L** | **100.00%** | **ENT_R** | **85.71%** |
| FUSI_L | 0.00% | FUSI_R | 0.00% |
| IP_L | 0.00% | IP_R | 0.00% |
| IT_L | 0.00% | IT_R | 0.00% |
| **ISTC_L** | **33.33%** | ISTC_R | 0.00% |
| **LOCC_L** | **95.24%** | **LOCC_R** | **100.00%** |
| LOF_L | 0.00% | LOF_R | 0.00% |
| LING_L | 0.00% | LING_R | 0.00% |
| MOF_L | 0.00% | MOF_R | 0.00% |
| MT_L | 0.00% | MT_R | 0.00% |
| **PHG_L** | **33.33%** | PHG_R | 0.00% |
| PARC_L | 0.00% | PARC_R | 0.00% |
| POPE_L | 0.00% | POPE_R | 0.00% |
| PORB_L | 0.00% | PORB_R | 0.00% |
| PTRI_L | 0.00% | PTRI_R | 0.00% |
| PERI_L | 0.00% | PERI_R | 0.00% |
| PSTC_L | 0.00% | PSTC_R | 0.00% |
| PC_L | 0.00% | PC_R | 0.00% |
| PREC_L | 4.76% | PREC_R | 0.00% |
| PCUN_L | 0.00% | PCUN_R | 0.00% |
| **RAC_L** | **71.43%** | RAC_R | 9.52% |
| RMF_L | 0.00% | RMF_R | 0.00% |
| SF_L | 0.00% | SF_R | 0.00% |
| SP_L | 0.00% | SP_R | 0.00% |
| **ST_L** | **100.00%** | **ST_R** | **100.00%** |
| SMAR_L | 14.29% | SMAR_R | 0.00% |
| **FP_L** | **71.43%** | **FP_R** | **28.57%** |
| TP_L | 0.00% | TP_R | 0.00% |
| TT_L | 0.00% | TT_R | 0.00% |
| INS_L | 0.00% | INS_R | 0.00% |
