# Supplementary material for: Construction of Individual Morphological Brain Networks with Multiple Morphometric Features
Source: Front Neuroanat. 2017 Apr 25;11:34. doi: 10.3389/fnana.2017.00034 (PMC5403938; doi:10.3389/fnana.2017.00034)
Supplement: Supplementary file 3 [file Table3.DOCX]

Table 3. The number of subjects that supports each node as a hub. The bold denotes the node with over 20% of subjects owned as a hub.

| **Regions** | **Proportion of subjects** | **Regions** | **Proportion of subjects** |
| --- | --- | --- | --- |
| BSTS_L | 10.91% | BSTS_R | 9.09% |
| CAC_L | 10.91% | CAC_R | 10.91% |
| **CMF_L** | **38.18%** | **CMF_R** | **27.27%** |
| CUN_L | 1.82% | CUN_R | 12.73% |
| **ENT_L** | **38.18%** | **ENT_R** | **25.45%** |
| FUSI_L | 14.55% | FUSI_R | 14.55% |
| IP_L | 5.45% | IP_R | 9.09% |
| **IT_L** | **21.82%** | IT_R | 0.00% |
| **ISTC_L** | **27.27%** | ISTC_R | 16.36% |
| **LOCC_L** | **32.73%** | **LOCC_R** | **38.18%** |
| LOF_L | 18.18% | LOF_R | 16.36% |
| LING_L | 14.55% | LING_R | 9.09% |
| MOF_L | 3.64% | MOF_R | 16.36% |
| MT_L | 7.27% | MT_R | 14.55% |
| **PHG_L** | **23.64%** | **PHG_R** | **29.09%** |
| PARC_L | 3.64% | PARC_R | 12.73% |
| POPE_L | 5.45% | POPE_R | 5.45% |
| PORB_L | 3.64% | PORB_R | 14.55% |
| PTRI_L | 10.91% | PTRI_R | 10.91% |
| PERI_L | 12.73% | PERI_R | 9.09% |
| PSTC_L | 10.91% | PSTC_R | 5.45% |
| PC_L | 14.55% | PC_R | 10.91% |
| PREC_L | 7.27% | PREC_R | 12.73% |
| PCUN_L | 7.27% | PCUN_R | 7.27% |
| **RAC_L** | **23.64%** | RAC_R | 16.36% |
| RMF_L | 5.45% | RMF_R | 12.73% |
| SF_L | 10.91% | SF_R | 12.73% |
| SP_L | 7.27% | SP_R | 7.27% |
| **ST_L** | **54.55%** | **ST_R** | **43.64%** |
| SMAR_L | 14.55% | SMAR_R | 9.09% |
| **FP_L** | **23.64%** | FP_R | 14.55% |
| TP_L | 9.09% | TP_R | 5.45% |
| TT_L | 12.73% | TT_R | 5.45% |
| INS_L | 1.82% | INS_R | 16.36% |
