# Supplementary material for: Construction of Individual Morphological Brain Networks with Multiple Morphometric Features
Source: Front Neuroanat. 2017 Apr 25;11:34. doi: 10.3389/fnana.2017.00034 (PMC5403938; doi:10.3389/fnana.2017.00034)
Supplement: Supplementary file 4 [file Table4.DOCX]

Table 4. The ICC of *C_p_*, *L_p_*, and BC at each sparsity. The bold denotes the insignificant results.

|  | ***C_p_*** | | ***L_p_*** | | **BC** | |
| --- | --- | --- | --- | --- | --- | --- |
| **Sparsity** | **ICC** | ***p*** | **ICC** | ***p*** | **ICC** | ***p*** |
| 0.2 | 0.825 | 3.77E-03 | 0.909 | 2.03E-04 | 0.787 | 8.16E-03 |
| 0.21 | 0.851 | 1.92E-03 | 0.809 | 5.30E-03 | 0.792 | 7.49E-03 |
| 0.22 | 0.866 | 1.21E-03 | 0.863 | 1.33E-03 | 0.782 | 9.06E-03 |
| 0.23 | 0.878 | 7.85E-04 | 0.837 | 2.75E-03 | 0.795 | 7.08E-03 |
| 0.24 | 0.894 | 4.05E-04 | 0.801 | 6.23E-03 | 0.71 | 2.55E-02 |
| 0.25 | 0.9 | 3.12E-04 | 0.882 | 6.76E-04 | 0.775 | 1.02E-02 |
| 0.26 | 0.9 | 3.09E-04 | 0.882 | 6.71E-04 | 0.787 | 8.20E-03 |
| 0.27 | 0.859 | 1.47E-03 | 0.858 | 1.55E-03 | 0.817 | 4.47E-03 |
| 0.28 | 0.862 | 1.35E-03 | 0.845 | 2.21E-03 | 0.808 | 5.48E-03 |
| 0.29 | 0.864 | 1.26E-03 | 0.824 | 3.83E-03 | 0.803 | 6.00E-03 |
| 0.3 | 0.835 | 2.89E-03 | 0.81 | 5.19E-03 | 0.8 | 6.45E-03 |
| 0.31 | 0.793 | 7.38E-03 | 0.777 | 9.78E-03 | 0.773 | 1.05E-02 |
| 0.32 | 0.776 | 9.98E-03 | 0.76 | 1.30E-02 | 0.759 | 1.33E-02 |
| 0.33 | 0.709 | 2.61E-02 | 0.744 | 1.65E-02 | 0.747 | 1.58E-02 |
| 0.34 | 0.757 | 1.35E-02 | 0.754 | 1.41E-02 | 0.757 | 1.35E-02 |
| 0.35 | 0.78 | 9.26E-03 | 0.81 | 5.28E-03 | 0.811 | 5.16E-03 |
| 0.36 | 0.784 | 8.70E-03 | 0.872 | 9.88E-04 | 0.872 | 9.72E-04 |
| 0.37 | 0.792 | 7.45E-03 | 0.874 | 8.91E-04 | 0.876 | 8.32E-04 |
| 0.38 | 0.828 | 3.45E-03 | 0.843 | 2.37E-03 | 0.842 | 2.46E-03 |
| 0.39 | 0.878 | 7.97E-04 | 0.699 | 2.90E-02 | 0.699 | 2.92E-02 |
| 0.4 | 0.878 | 7.79E-04 | **0.631** | **5.64E-02** | **0.629** | **5.72E-02** |
